# Amyloid-β-Induced Neurotoxicity Modulates miR-98 and miR-200 Expression in SH-SY5Y Cells: A Step Toward Alzheimer’s Biomarker Discovery

**DOI:** 10.1007/s12035-025-04895-5

**Published:** 2025-04-07

**Authors:** Ezgi Keske, Ayyub Ebrahimi, Özlem Sağlam Uçar

**Affiliations:** 1 İstanbul Medeniyet University, Istanbul, Turkey; 2 Molecular Biology and Genetics, Haliç University, Istanbul, Turkey; 3 Present Address: Functional Gene Control, Laboratory of Medical Sciences, Medical Research Council, London, UK; 4 Department of Medical Biology, Faculty of Medicine, İstanbul Nişantaşı University, Istanbul, Turkey

**Keywords:** Alzheimer’s disease, Amyloid plaque, MicroRNA, Biomarker

## Abstract

Alzheimer’s disease (AD) is a progressive neurodegenerative disorder characterized by abnormal protein accumulation, with no effective, non-invasive early diagnostic tools currently available. MicroRNAs (miRNAs), essential for neuronal survival and function, have been implicated in AD neuropathology. This study investigates the potential of miRNAs as biomarkers for AD by assessing the expression levels of miRNAs relevant to amyloid toxicity. An AD model was developed in SH-SY5Y human neuroblastoma cells with adequate Aβ42 expression to analyze the involvement of miRNAs in AD diagnosis. ELISA, MTT assays, and Congo red staining were utilized to quantify qualitative and quantitative amyloid formation. The expression of miRNAs and related genes, particularly those targeting APP and β-secretase, was measured using quantitative real-time PCR. Amyloid toxicity was successfully induced, and an increase in amyloid levels and significant changes in Alzheimer’s related genes and targeted miRNAs were observed. Specifically, it was observed that miR-200a was upregulated and miR-98 was down-regulated in treated neuroblastoma cells. Notably, the altered expression patterns of these miRNAs showed a strong correlation with the pathological markers of AD, suggesting their potential as diagnostic indicators. Our findings enhance our understanding of AD mechanisms and offer insights into early diagnosis. Detecting AD in preclinical stages may enable earlier symptomatic intervention. In particular, dysregulation of certain miRNAs may play a role in neurodegenerative processes such as amyloid plaque formation in AD. miRNAs that respond to neurotoxic stimuli can be identified using in vitro models and confirmed by in vivo studies. These studies will help us understand both the development of noninvasive diagnostic tests and therapeutic approaches targeting miRNAs.

## Introduction

Alzheimer’s disease (AD) is a complex and progressive neurodegenerative disorder primarily characterized by cognitive decline and behavioral changes that significantly impair daily functioning. It is the most common cause of dementia, affecting millions of people worldwide and presenting substantial challenges for patients, families, and healthcare systems [1, 2]. The pathophysiology of AD is primarily marked by the accumulation of amyloid-beta (Aβ) plaques and hyperphosphorylated tau protein, which leads to neuroinflammation, synaptic dysfunction, and, ultimately, neuronal death [3, 4]. The disease typically begins in the hippocampus—an area crucial for memory—and gradually spreads to other parts of the brain, resulting in severe cognitive impairment [5].

As AD progresses, patients may exhibit a range of debilitating symptoms, including severe memory loss, language difficulties, and changes in personality, such as agitation and hallucinations. These symptoms not only affect the quality of life of individuals with AD but also impose significant emotional and financial burdens on caregivers and society [6]. Given the increasing incidence of AD due to the aging population, there is an urgent need for effective diagnostic tools and therapeutic interventions.

The diagnosis of AD often relies on clinical assessments, which include cognitive evaluations and the identification of characteristic behavioral symptoms. Advanced diagnostic techniques, such as positron emission tomography (PET) imaging and magnetic resonance imaging (MRI), are used to visualize brain changes associated with the disease. However, these methods have limitations, including potential false positives and the challenge of diagnosing AD in the absence of overt clinical symptoms. Studies have shown that neurodegenerative processes may commence 10 to 20 years before the onset of clinical manifestations, highlighting the need for early and reliable diagnostic approaches [7]. Consequently, research efforts have increasingly focused on identifying biological markers that can facilitate the early detection of AD.

Two hallmark features of AD pathology are the formation of amyloid plaques and neurofibrillary tangles (NFTs). Amyloid precursor protein (APP) undergoes proteolytic cleavage by β- and γ-secretases, leading to the generation of toxic amyloid-beta (Aβ) peptides that aggregate into insoluble plaques [8]. These aggregates disrupt neuronal communication and trigger inflammatory responses that contribute to synaptic and neuronal damage [9]. Moreover, the hyperphosphorylation of tau, a microtubule-associated protein, leads to its dissociation from microtubules and the formation of NFTs, which further exacerbate neurodegeneration [10].

Recent research has indicated that the complex interplay between Aβ and tau pathology is critical for the progression of AD. For instance, studies have shown that Aβ accumulation can induce tau hyperphosphorylation and aggregation, suggesting that targeting both pathways simultaneously may be a promising therapeutic strategy [11]. Moreover, the involvement of tau in synaptic dysfunction emphasizes the importance of understanding its role in AD pathology and exploring potential interventions that could mitigate its effects [12].

Given the ethical concerns surrounding invasive diagnostic procedures and the limitations of current clinical diagnostic methods, there has been a growing interest in the role of microRNAs (miRNAs) as non-invasive biomarkers for AD. These small, non-coding RNA molecules are known to regulate gene expression post-transcriptionally, influencing various biological processes, including neuronal development, differentiation, and synaptic plasticity [13]. Altered miRNA expression profiles have been associated with a range of neurological disorders, including AD, indicating their potential utility as biomarkers for early diagnosis [14, 15]. Recent studies have identified specific miRNAs that exhibit significant changes in expression in the context of AD, further supporting their role in disease pathogenesis [16, 17].

Research has suggested that miRNAs may reflect disease-specific patterns and that their dysregulation may play a critical role in the mechanisms underlying AD [18]. For instance, studies have highlighted the potential of miR-206, miR-132, and miR-146a in modulating amyloid and tau pathology, thereby influencing disease progression [19, 20]. Furthermore, the non-invasive nature of miRNA analysis through blood or cerebrospinal fluid samples offers a promising avenue for early detection and monitoring of AD.

In this study, we aimed to investigate the potential of specific miRNAs as biomarkers for Alzheimer’s disease by inducing amyloid toxicity in cellular models and assessing the expression levels of miRNAs previously implicated in AD pathogenesis. By elucidating the relationship between miRNA expression and Aβ toxicity, we hope to contribute to the development of non-invasive diagnostic strategies that facilitate early intervention in Alzheimer’s disease.

## Material and Methods

### Cell Culture

SH-SY5Y human neuroblastoma cells were obtained from the Department of Medical Biology, Faculty of Cerrahpaşa Medicine, Istanbul University. SH-SY5Y cells were cultured in DMEM (Thermo Fisher) containing 10% fetal bovine serum (FBS) (Thermo Fisher), 1% NEAA (Thermo Fisher), and 1% penicillin/streptomycin (Thermo Fisher) at 37 °C, 5% CO_2_, and a humid environment that allowed cells to proliferate well. The cells’ medium was replaced every 3 days. The cells were subcultured when they reached 70–80% confluency.

### Preparation of Aβ42 Peptide and Aβ42 Exposure

The Aβ (1–42) protein fragment (Sigma-Aldrich) stock (1 mg) was dissolved in dimethyl sulfoxide (DMSO) as per the protocol to a final concentration of 2.5 mM. The product with a molecular weight of 4514.04 g/mol, provided by the company, was dissolved in DMSO (Sigma-Aldrich) as indicated in the information sheet. The prepared Aβ42 peptide stock solution was aliquoted and stored at − 80 °C.

Cells were cultured in 6-well plates for Aβ42 treatment. Aβ42 peptide was applied to the cells at a final concentration of 5 µM. Then, the cells were incubated for 48 h. At the end of 48 h, the medium containing the Aβ42 peptide was removed from the cells and incubated for 48 h with fresh medium.

Congo Red was used to stain the amyloid residues. The Congo Red Stain Kit (Highman) procedure was used for staining. After the cells were treated with Aβ42 in 6-well plates, the medium was removed, and the cells were fixed with absolute ethanol. Congo red dye solution was added to the fixed cells and waited for 5–6 min then submerged in potassium hydroxide for 15–20 s. After washing the wells, Haemalum Mayer’s solution was added and waited for 5–6 min. The wells were washed and allowed to dry. The amyloid residues were examined under an inverted microscope.

### Cell Viability Assay

MTT (3-(4,5-dimethylthiazol2-yl-2,5-diphenyl tetrazolium bromide, Biomatik) analysis was performed to measure the doubling times of the cells and the toxic effect of Aβ42 protein on the cells. Cells were passaged into 96-well plates. Aβ42 peptide was applied to the cells at a final concentration of 5 µM. Then, the cells were incubated for 48 h. 0.5 mg/mL MTT solution was added and was incubated for 3–3.5 h. After the blue formazan crystals were seen, the wells were washed with PBS (Thermo Fisher) and incubated with DMSO (Sigma) in the dark for 10 min. The measurement was performed at 595 nm.

### Enzyme-Linked Immunosorbent Assay (ELISA)

After the treatment of cells with Aβ42, supernatants were collected, and quantitative analysis of the Aβ42 protein was performed with the Invitrogen ™ Human Aβ42 ELISA Kit. Standards were prepared and loaded into the 96-well plates with samples. Human Aβ42 detection antibody was added to the wells, and the plate was incubated for 3 h in an orbital shaker. At the end of 3 h, the wells were washed, and the Anti-Rabbit IgG HRP solution was added and incubated for 30 min. The plate was washed again and incubated with Stabilized Choromgen for another 30 min in the dark. By adding Stop Solution, absorbance values were measured at 450 nm.

### Total RNA Extraction, cDNA Synthesis, and Quantitative Real-Time Polymerase Chain Reaction (qRT-PCR)

Cells treated with Aβ42 protein and control cells were harvested for total RNA extraction. Total RNA was isolated from cells using the NucleoSpin® RNA kit (Machery-Nagel) following the supplier’s guidelines. To isolate total RNA, we first collected cells with 0.05% trypsin (Thermo Fisher) and then spun them down in a centrifuge to separate them from the liquid. After discarding the liquid, we added a lysis buffer containing Beta-mercaptoethanol (β-ME) as a reducing agent to lyse the cells and mixed well. The mixture was then placed in a special filter tube and spun again to separate the contents. Next, we added 70% ethanol to the filtered liquid and mixed it before passing it through a new filter, followed by another quick spin. After discarding the liquid, we added a desalting buffer to the filter and spun it once more. We then introduced a reaction mixture to degrade any remaining DNA and incubate it at room temperature for about 15 min before washing. Finally, we added RNase-free water and did one last spin. After removing the filter, we used a MultiSkan GO μDrop device (Thermo Scientific) to measure the concentration of the total RNA we had isolated. Isolated RNAs were converted to cDNA (MystiCq™ microRNA cDNA Synthesis Mix, Sigma-Aldrich). To create complementary DNA (cDNA) from total RNA, we used the ProtoScript® First Strand cDNA Synthesis Kit. Relevant solutions in the kit were added to 1 µg of the total RNA, and cDNA synthesis was performed according to the given temperatures and times in the thermal cycler 70 °C for 5 min, 42 °C for an hour, and 80 °C for 5 min. Synthesized cDNAs were stored at − 20 °C for future analysis.

To study Alzheimer’s disease at the cellular level, real-time polymerase chain reaction (RT-PCR) was used to compare the expression of AD-associated genes, focusing on APP due to its ability to induce Aβ42 formation. We followed the SensiFAST™ SYBR® No-ROX Kit (Bioline) guidelines for the PCR process. Equal amounts of cDNA were used, and the gene expression levels were measured against a reference gene. The PCR involved preparing a mixture, followed by a heating step at 95 °C for 2 min, and then 40 cycles of 5 s at 95 °C, 10 s at 60 °C, and 20 s at 72 °C, concluding with a melt temperature adjustment. Data was determined via the 2^–∆∆CT^ method. The results were expressed as fold differences in gene expression between Aβ42 treatment cells and non-treated conditions.

### miRNA Extraction, cDNA Synthesis, and Quantitative Real-Time Polymerase Chain Reaction (qRT-PCR)

Cells treated with Aβ42 protein and non-treated control cells were harvested by trypsin for micro RNA extraction. miRNAs were isolated from cells using the mirPremier® micro RNA Isolation Kit (Sigma-Aldrich) following the manuals. To do that, cells were first washed with Hank’s Balanced Salt Solution and then lysed with a lysis mixture containing β-ME in the kit. After treating them with the lysis solution, the mixture was spun in a centrifuge at high speed for 5 min. Then, an equal amount of 100% ethanol was added to the remaining liquid and mixed thoroughly. This mix was transferred to a filter tube and centrifuged again for 30 s. The filtered liquid (flowthrough) was discarded, and more ethanol was added before another quick spin to remove any remaining liquid. Next, a diluted solution was introduced to the filter and centrifuged one last time. After discarding the flowthrough, the filter was spun once more without any added solution to ensure it was dry. Then, a specific amount of elution solution was added to the filter, and after waiting for a minute at room temperature, it was centrifuged again. This step was repeated once more before disposing of the filter. Finally, the concentration of the extracted miRNA was measured using the MultiSkan GO μDrop device (Thermo Scientific). To create complementary DNA (cDNA) from microRNA, the MtstiCq™ microRNA cDNA Synthesis Mix Kit (Sigma-Aldrich) was used. According to the guidelines, miRNAs were mixed and gently swirled. Then, the miRNA mixture was incubated at 37 °C for an hour, followed by a quick 5-min heat at 70 °C. After that, we added the given additional components and incubated them at 42 °C for 20 min, finishing with another 5-min heating at 85 °C. Finally, we stored the newly synthesized cDNA in the freezer at − 20 °C for future analysis.

Expression levels of candidated miRNAs associated with the AD (miR-16, miR-223, miR-98, miR-9-3p, miR-9-5p, miR-29a, and miR-200a) were measured by quantitative RT-PCR with MystiCq™ microRNA SYBR® Green qPCR ReadyMix™ (Sigma-Aldrich). The following PCR program was used to measure miRNA levels (2 min at 95 °C, followed by 40 cycles of 95 °C for 5 s, 63 °C for 15 s, and 70 °C for 15 s, ending with a melt temperature step. Data was determined via the 2^–∆∆CT^ method. The results were expressed as fold differences in miRNA expression between Aβ42 treated and non-treated cells.

### Statistical Analysis

Statistical analysis was performed using GraphPad Prism 8.0.2. Differences between the two groups were determined using a one-way analysis of variance (ANOVA) or Student’s *t*-test. The data obtained as a result of real-time PCR applied to determine the expression levels of genes and miRNAs were evaluated in the analysis software of the BIO-RAD CFX ConnectTM Real-Time PCR device, and the significance values obtained from this program were used. The results are presented as the mean ± standard deviation (SD). The data with a *P*-value of *P* ≤ *0.05* were considered statistically significant.

## Results

### Microscopic Insights into Amyloid Beta-Induced Cellular Toxicity

**Fig. 1:**
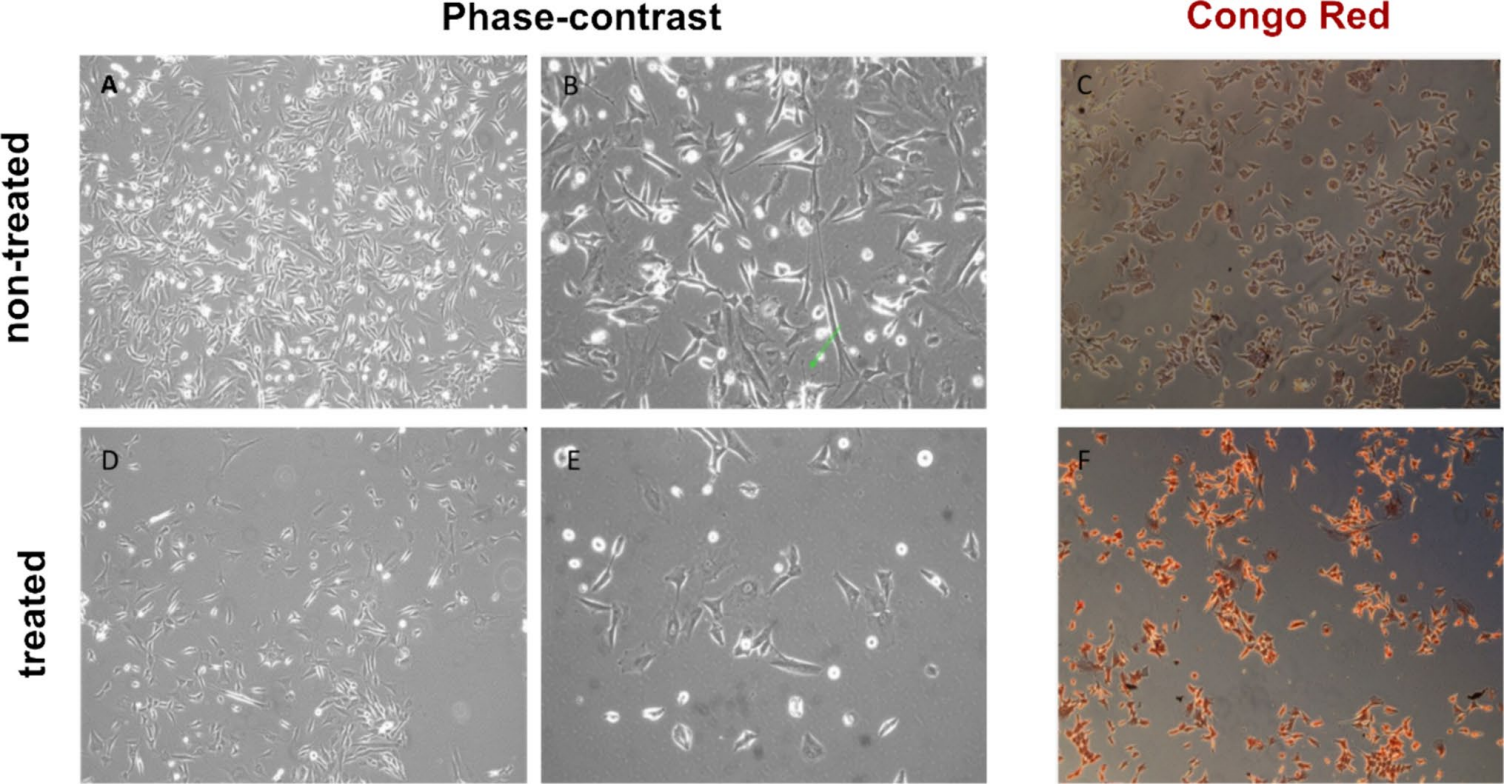
Aβ42 treatment and Congo red staining in SH-SY5Y cells. **A**, **B** Microscopic images of non-treated control cells. **C** Congo red staining in non-treated cells. **D**, **E** Microscopic images of Aβ42-treated cells. **F** Congo red staining in Aβ42-treated cells. Magnifications: × 5 (**C**, **F**), × 10 (**A**, **D**), × 20 (**B**, **E**)

Cells that were not treated with the Aβ42 peptide were observed to be denser than treated cells. In addition, it was observed that the dendrite-like structures were more distinct in the non-treated control group. The existence of amyloid-beta accumulation in cells was supported using Congo red. Congo red staining showed that the most toxic form of extracellular amyloid plaques formed inside the cells and emerged into the extracellular matrix (Fig. 1).

### Impact of Amyloid Beta on Cell Viability Through MTT Analysis Over Time

MTT analysis was used to determine both the doubling time and the viability of the Aβ42-treated and non-treated cells. The analysis aided in optimizing the Aβ42 peptide application time. It was discovered that SH-SY5Y cells doubled their amount in roughly 44 h following the values obtained through the MTT assay.

**Fig. 2:**
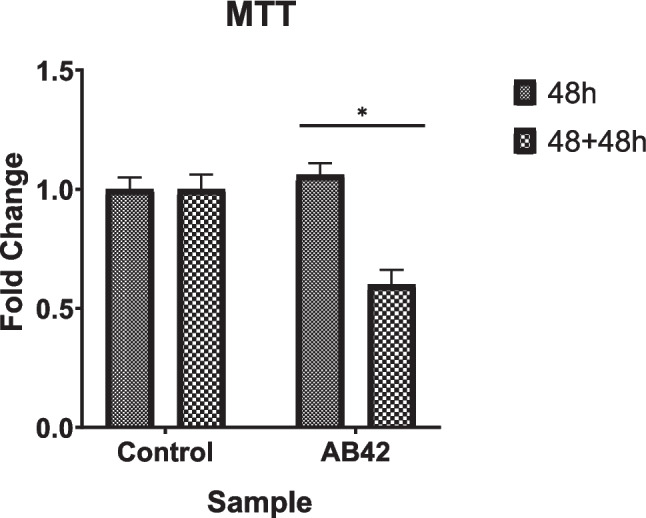
Cell viability assay following Aβ42 treatment. Treatment with Aβ42 resulted in a significant decrease in cell viability, with a 40% reduction observed after 96 h (48 + 48 h) (*p ≤ *0.05*). This indicates a marked cytotoxic effect of Aβ42 on cellular health throughout the experiment

After the Aβ42 treatment, MTT analyses of SH-SY5Y cells were performed to measure the cell viability. In the measurement performed 48 h after the protein application, when comparing the viability of Aβ42 treated SH-SY5Y cells to those that were not applied, we observed an increase of approximately 1.06. After 48 h of Aβ42 treatment, the medium was renewed, and the viability analysis of the cells that were left to stand for 48 h was determined by MTT. When we compared the Aβ42-treated cells to the non-treated cells, we observed that they decreased by approximately 0.6 in SH-SY5Y cells (Fig. 2).

### ELISA Analysis of Protein Alterations Induced by Amyloid Treatment

ELISA analysis was performed for the quantitative measurement of the amount of Aβ42 protein. Aβ42 was applied to the cells in 6-well culture plates in an appropriate amount to the surface area. The medium on the cells was collected for ELISA. Thus, the amount of Aβ42 protein was measured comparatively by ELISA between cells exposed to a medium containing Aβ42 protein and non-treated control groups.

**Fig. 3:**
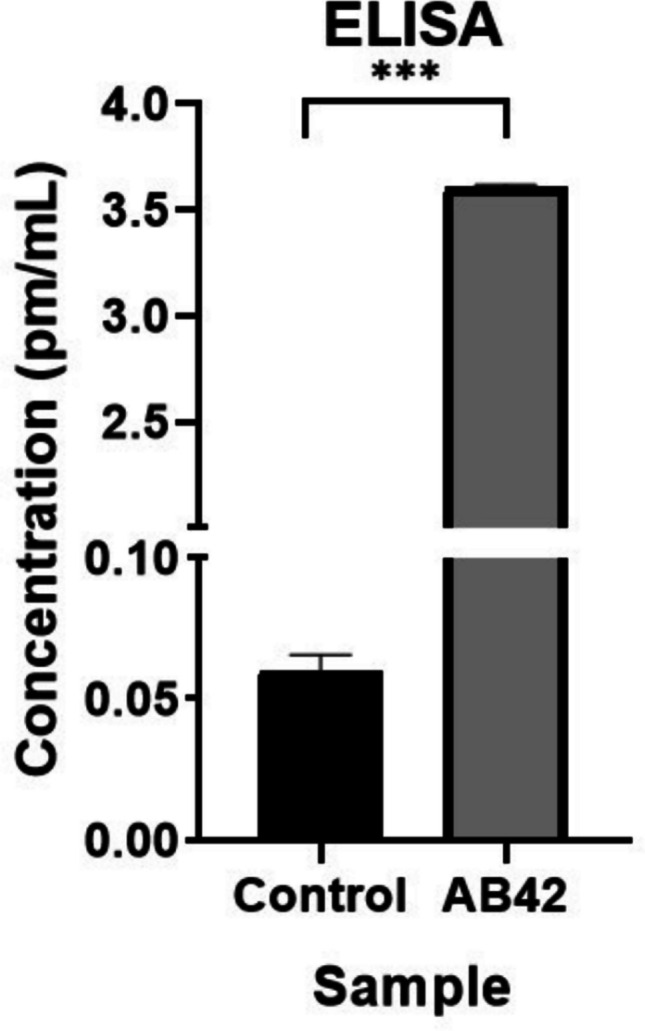
ELISA measurement of amyloid beta 42 (Aβ42) treated cells demonstrates a significant increase in amyloid levels. Treatment with Aβ42 results in an approximately 60-fold increase in amyloid accumulation compared to non-treated controls (***p ≤ *0.0001*). This indicates a robust response to Aβ42 treatment in the measured samples

After the application of the Aβ42 protein to the cells with a final concentration of 5 µM, ELISA measurement was performed on the supernatants collected without passaging of the cells. As a result of this measurement, it was determined that the treated cells contained much more Aβ42 protein than the control cells (Fig. 3).

### Quantitative Expression Analysis of Genes and Associated miRNAs by qPCR

**Fig. 4:**
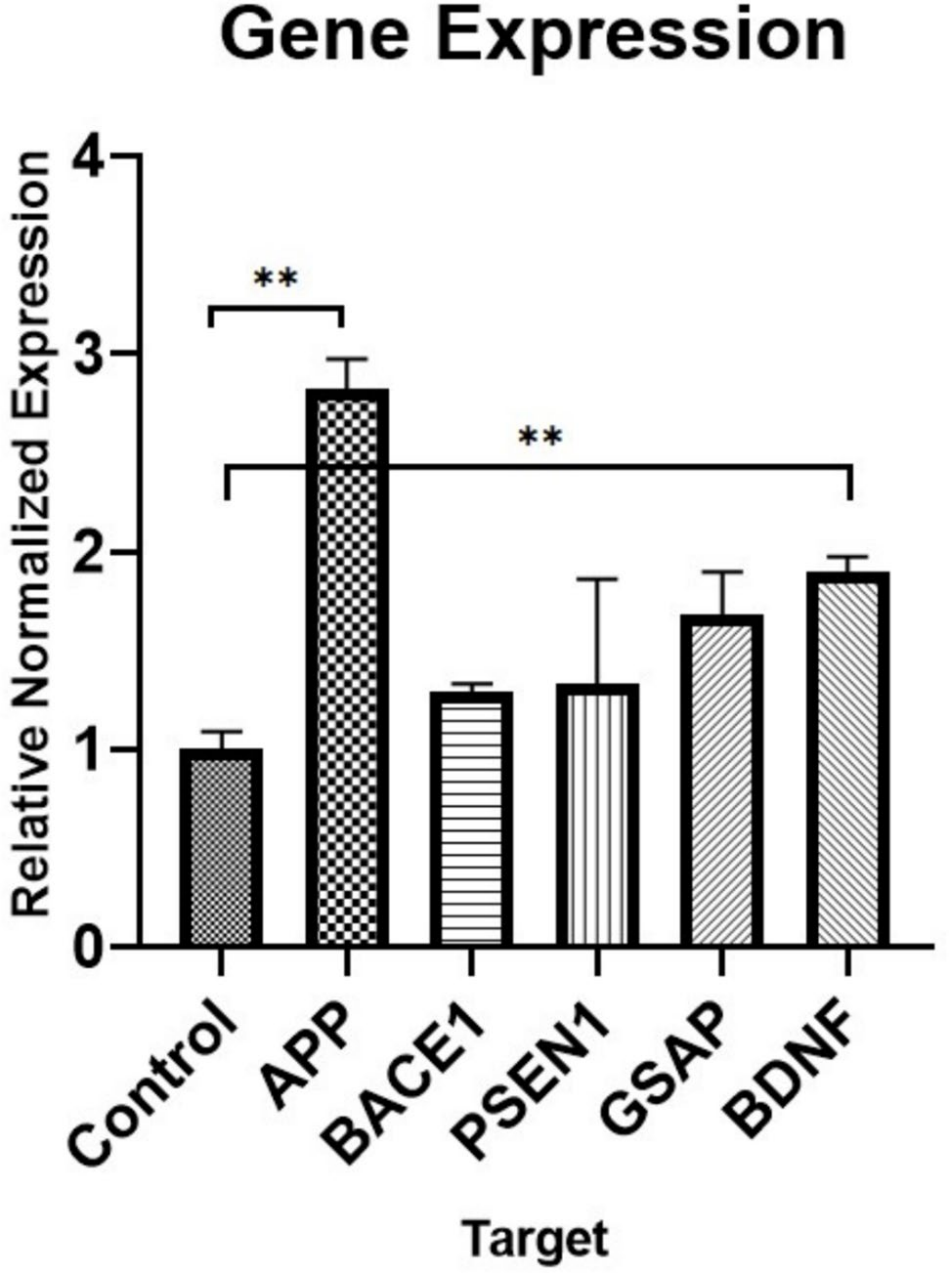
qRT-PCR results following Aβ42 treatment in SH-SY5Y cells. Treatment with Aβ42 resulted in a significant 2.8-fold increase in APP expression and a 1.9-fold increase in BDNF expression (**p ≤ 0.005). No statistically significant changes were observed in the expression levels of other examined genes. These findings indicate a specific upregulation of APP and BDNF in response to Aβ42 treatment

The expression level of AD-related genes following the Aβ42 protein administration to SH-SY5Y cells was assessed with qRT-PCR. For that, RNA was isolated and cDNA was synthesized in Aβ42-treated cells and non-treated control groups. The analysis showed increased expression levels for all genes tested (Fig. 4).

The quantitative analyses performed with miR-16, miR-223, miR-98, miR-9-3p, miR-9-5p, miR-29a, and, miR-200a for the preliminary study. miR200a and miR98 were selected due to their statistical significance for further investigation.

**Fig. 5:**
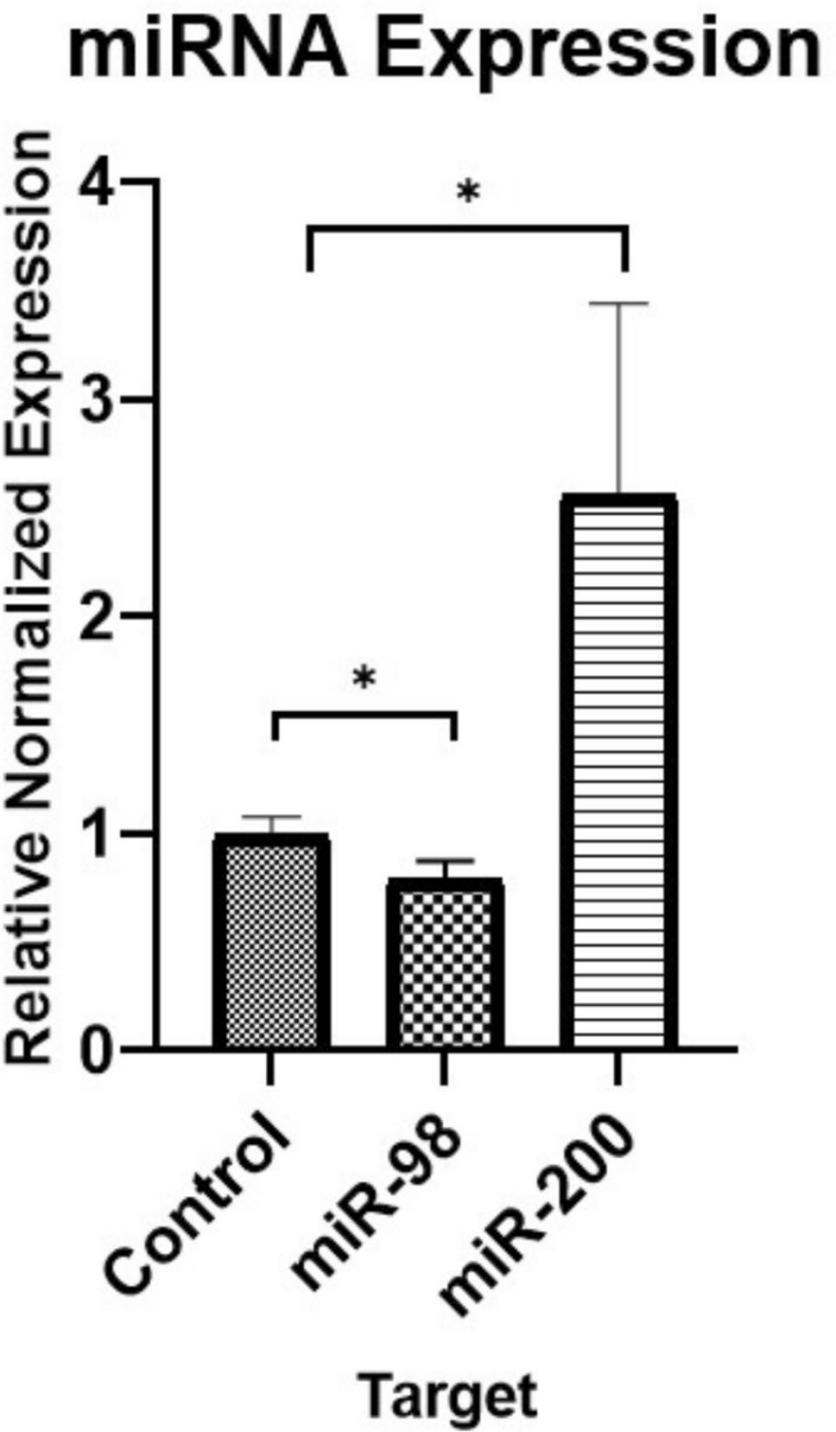
Quantitative analysis of miR-200a and miR-98 expression in SH-SY5Y cells. Data shows a significant 2.6-fold increase in miR-200a expression (*p ≤ 0.05), alongside a notable 0.2-fold decrease in miR-98 expression. In contrast, changes in the expression levels of other miRNAs were not statistically significant. These findings suggest a potential regulatory role of miR-200a and miR-98 in SH-SY5Y cells

The expression level of miRNAs associated with the abovementioned genes was examined by qRT-PCR in SH-SY5Y cells (Fig. 5). As a result of this quantitative analysis, a statistically significant increase in miR-200a was observed in SH-SY5Y cells treated with Aβ42 protein (*p ≤ 0.05). The decrease in miR-98 showed statistical borderline significance (0.05 ≤ *p* ≤ 0.1).

## Discussion

Alzheimer’s disease is the most common type of dementia worldwide. The obstacle to the desired progress in the diagnosis and treatment stages of the disease is the inability to understand the relations between the aggregates themselves and each other. There are so many knots that need to be resolved. One of the knots in the mechanism is the accumulation of amyloid-beta plaques, which is one of the main causes of the disease. The detection of a non-invasive biomarker is critical in the prognosis and follow-up of the disease, especially for early diagnosis. Long before clinical symptoms appear, pathological changes in the brains of Alzheimer’s patients begin. The lack of specific biomarkers is one of the biggest factors in the failure of early diagnosis of the disease. The ideal biomarker should be specific and easy to measure, describing the neurodegenerative process before cognitive deterioration. Amyloid β42 and Tau proteins have been identified as cerebrospinal biomarkers. Amyloid β oligomers are also candidates for synaptic markers [4, 21]. However, the markers that have been found are insufficient to detect the disease at an early stage.

Synaptic loss is a hallmark of AD, primarily attributed to insoluble amyloid accumulation [22]. According to the amyloid hypothesis, amyloid plaques, resulting from amyloid precursor protein (APP) cleavage, play a central role in AD pathogenesis [23]. This study aimed to explore AD-associated genes, miRNA levels, and potential biomarkers by inducing amyloid toxicity in SH-SY5Y human neuroblastoma cells, which may generate amyloid themselves.

Morphological analysis of SH-SY5Y cells treated with Aβ42 revealed significant structural changes, including reduced dendrite-like projections and altered cell shapes (Fig. 1). Microscopy and MTT assays demonstrated that Aβ42 is cytotoxic, as evidenced by statistically significant reductions in cell viability at 48 and 96 h (Fig. 2). The doubling time of SH-SY5Y cells was approximately 48 h. To assess the increase in amyloid production and its associated toxicity, we selected the 48-h and 96-h time points for observation. While simulating toxicity in cells with Aβ42, various approaches were attempted and the most effective one was chosen within the experimental protocol. For example, after applying Aβ42 to the cells, they have progressed three passages. Cells that progressed three passages were stained with Congo red almost at the same as those studied immediately after the Aβ42-treated cell. The findings were confirmed with the ELISA test. In this situation, it was assumed that the cells would not maintain their toxicity for an extended time, but the increased Aβ42 impact would. Furthermore, as compared to other cells, SH-SY5Y cells are capable of producing amyloid toxicity when given the right conditions, which is thought to be proof of cells' feedback on amyloid accumulations.

Interestingly, MTT assays indicated a transient increase in cell proliferation shortly after the Aβ42 application, which declined over time. This initial proliferation may reflect a protective response to amyloid buildup, consistent with previous reports [24]. However, prolonged exposure to Aβ42 disrupted synaptic communication and exacerbated neuronal damage, underscoring its toxic effects [25].

Qualitative Congo red staining (Fig. 1) confirmed amyloid accumulation, both intracellularly and in the extracellular matrix, with a modest quantity of endogenous amyloid in untreated SH-SY5Y cells as predicted [22], highlighting the amyloidogenic pathway involving APP cleavage by β- and γ-secretases. Alzheimer’s disease has been associated with many genes, both directly and indirectly. APP and presenilin-1 (PSEN1), genes strongly associated with AD, exhibited increased expression in response to Aβ42, aligning with their roles in amyloid production [26]. In the qRT-PCR analysis, an increase in the expression of APP and PSEN1 genes was observed in SH-SY5Y cells with toxicity compared to controls. Brain-derived neurotrophic factor (BDNF), a critical neurotrophic gene, showed reduced expression, consistent with observations in AD patients’ hippocampal and cortical regions, which are responsible for learning and memory [27]. The γ-secretase activating protein (GSAP) gene, which influences Aβ production, also demonstrated changes that mirrored amyloid toxicity [28–35]. Taking the PCR results into account, it is feasible to conclude that the supplied Aβ42 increases toxicity in the cells and that healthy cells respond defensively as an initial reaction to this rise due to the short-term administration. This elevation in gene expression levels indicates that Aβ42 induces toxicity in cells and triggers AD-related gene expressions. The increase in APP in SH-SY5Y cells suggests that we are mimicking the disease in the cells and establishing feedback for amyloid deposition. An increase in the expression of AD-related genes was observed in experimental data following amyloid exposure. Additionally, miR expressions were analyzed after amyloid modeling had been established in the cells.

miRNAs are molecules that regulate biological processes. By cutting or suppressing the target mRNA, they participate in post-transcriptional regulation. miRNAs can bind to the target mRNA numerous times during this regulation, and each mRNA can be controlled by multiple miRNAs [29–36]. Experimental research has shown that the 5′ region of miRNA is the region where the activity begins and specific binding to the target [26]. It is thought that miRNA has important roles in neuronal development, differentiation, and synaptic plasticity of neurons, and problems occurring in miRNAs may cause diseases such as central nervous system diseases like Alzheimer’s disease and Huntington’s disease [37, 38]. The studies in this area assume that because dysregulation of particular miRNAs has been discovered in Alzheimer’s disease, this aberration may play a role in the pathology of the disease, and so miRNAs may be noninvasive and sensitive biomarkers [29, 30, 32]. In this context, we used qRT-PCR to look at the expression levels of certain miRNAs that we correlated with disease-related genes.

The level of miR-9 is low in Alzheimer’s patients, and there is a lot of evidence that they are linked to BACE1 in particular. BACE1 has been demonstrated to be suppressed by miR-29a in the β-secretase molecular pathway, which is involved in the enzymatic cleavage of APP [20]. It has been observed that miR-29a has a negative effect on the expression of DNA methyltransferase, which contributes to neural proliferation by regulating BDNF expression [34]. miR-16 has been identified as a potential inhibitor of APP and BACE1 that is effective in Aβ peptide production. A relationship was found between misfolded amyloid and miR-16 in Alzheimer’s patients [32–35]. In addition, in a study on SH-SY5Y cells with increased miR-16 levels, it was observed that BDNF expression decreased significantly [33, 34]. miR-9-5p, miR-9-3p, and miR-16 expressions were decreased and miR-29a expression level was increased in SH-SY5Y cells, but the increases and decreases were not found statistically significant.

These findings reinforce the role of miRNAs as regulators of AD-related genes, supporting their use as biomarkers. Notably, miR-98 and miR-200a showed significant expression changes, suggesting their potential utility in AD diagnosis. The study also validates the amyloid cascade hypothesis, demonstrating how amyloid metabolism perpetuates its own production, further exacerbating disease pathology.

To summarize, in the new phase of AD research, SH-SY5Y cells have begun to be used as cells that can produce amyloid-beta, albeit at small levels. Some SH-SY5Y cells were treated with Aβ42 protein, while others were left as controls, and all cells were exposed to the same process at the same time to imitate AD in cells. The Aβ42 protein triggered its production by producing feedback in SH-SY5Y cells, according to ELISA analyses and staining methods. qRT-PCR was used to look for significant connections between the genes linked with the disease and the expressions of their related miRNAs after modeling the disease.

Although numerous sources in the literature have mentioned miR-98 as a potential biomarker, there is not much information about miR-200a. MiR-98 and miR-200a expression level alterations with significant statistical significance imply that they may be employed as biomarkers for Alzheimer’s disease.

When the active roles of miRNAs in the molecular mechanism of the cell are evaluated, it is clear that their definition as biological markers has risen as a result of recent studies. miRNAs appear to be regulatory on genes that directly or indirectly impact the metabolism of the disease, according to the current research. Furthermore, the amyloid cascade hypothesis was once again validated considering the findings, and it was demonstrated that amyloid metabolism triggers its production and that its greater amount is significant in Alzheimer’s disease. Therefore, our research contributes to the understanding of Alzheimer’s disease’s mechanism and diagnosis. By diagnosing the disease in the stages before the appearance of clinical symptoms, it will be feasible to begin symptomatic treatment at an early stage or take required actions in light of developments.

## Data Availability

No datasets were generated or analysed during the current study.
